# The Presidential Address, Wisconsin Society of Veterinary Graduates1Read before the meeting of the Society, at Madison, February 5, 1897.

**Published:** 1897-06

**Authors:** G. Ed. Leech

**Affiliations:** Milwaukee, Wis.


### THE PRESIDENTIAL ADDRESS, WISCONSIN SOCIETY OF VETERINARY GRADUATES.[1]

By G. Ed. Leech,
MILWAUKEE, WIS.

When the gavel fell calling the meeting to order it bade you all welcome to the field of labor, not as in the quarries or on the farm, or in the field of mechanics, but unceasing labor in the most interesting of all fields to each one whose chosen profession is veterinary science.

You are not expected to work upon the foundation, for that was laid, so far as history gives us information, in the days of the greatest of all men—intellectually—the wise King Solomon; and with the

[1] Read before the meeting of the Society, at Madison, February 5, 1897.

history of the beginning of the work in the State of Wisconsin, I take it, you are all familiar. But it is our duty now to aid in building the superstructure, and let it be our one thought to do our work so thoroughly that it will withstand the adverse criticism of ages to come. By a united and persistent effort much has been accomplished: no country has a better system of food-inspection; our colleges have unanimously adopted a three-years' course, with a higher standard of qualification; and constant improvement is shown by the journals, which are excelled by none. Nearly every State with important agricultural interests has enacted stringent laws, and ere the present session of our Legislature ends this State will be in line with the rest that are looking to better their condition in the matter of veterinary science. I would that the motto of our Society might be "Patriotism and Coöperation." There seems to be a decrease of patriotic and coöperative spirit all over the country in our profession. Patriotism has come generally to be considered and interpreted as a willingness to fight and die for one's country and its institutions. That definition answers very well for patriotism in time of war, but it is deficient in that it allows no room for patriotism in time of peace. We should consider that a very weak specimen of conjugal fidelity which impelled a man to care for his wife and devote himself to her necessities only on occasions when she was threatened by ruffians. A husband's love has its sphere of devotion at all times and in all situations; so has patriotism. If a man loves his profession and is true to her societies and affectionately concerned for their standing and permanence, he will be all the time doing something in her behalf. What our profession needs most is men who will live for her, not simply die for her, and live for her every day of their lives. A member of any profession has no more right to be neglectful of its interests than a parent or child has to be neglectful of the interests of the family of which he is a member. There is the same condition of responsibility involved in both cases, and the man who neglects either is rightly looked upon as a shirk. What is needed in both cases to bring about a condition of success is coöperation. Elect whom you will as the officers of your society, and unless they receive the hearty coöperation and support of the members they are in the position of the editor of a first-class journal who has the best selections and contributions from the pens of the most eminent writers, but has no subscribers. In both cases the result will be failure. In no case can any man or society expect to rise above their ability, and all depends upon coöperation.

What I wish to convey to your minds in this connection is this, we are composed practically of a number of young men, and what is the use of being young men with warm blood in our veins and live brains in our skulls, if in matters of so vast an import as these we have to be wound up every six or twelve months, in order to make us strike when the hour comes, and to be held over a slow fire of agitation before every meeting in order to make us hot enough to be passionately interested in the welfare of our societies, and to speak and act with an earnestness that will secure that welfare.

Our Society since its incorporation has not grown in numbers befitting the profession in the State, neither has it been favored with an annual attendance creditable to its membership; but, with harmony and coöperation once firmly established among the members as their motto, our ranks will soon be increased. If there is any young man aware of the aim and purposes of our Society and free from the influence of our wily antagonists, who can survey the situation without feeling a desire to enter into its membership, may you all have compassion for him. Our mission in life is to furnish the best and most scientific treatment to a class of patients who are unable to express their gratitude to us in well chosen and flowery language for the results obtained by our achievements of learning; but among those who employ us it is coming to be a recognized fact that they must look to us for that achievement, and it is incumbent upon us not to betray that confidence.

With patriotism and coöperation for our motto we will be enabled to place within the walls of our superstructure that kind of material that will redound to our credit and wider influence. My only concern is that those who are thinking about the superstructure of earnest and far-reaching intelligence and an elevated and vigorous society may forget the fact that much depends upon their own personal efforts; they should with sagacious and conscientious fidelity devote themselves to building up their own possibilities as a fortress against the unscrupulous practitioner and wily politician. Our Society's usefulness will cease the moment we allow its influence to be used in other than a professional line. We are not organized for any other purpose, and we should use our efforts in this line only. Every department of food-supply in the State should be under our control, and our members should make untiring and unceasing efforts to accomplish this end. The dairy-farms and their products should be inspected, the milk sent from the country to the city should be guaranteed free from contagion, and the attendants upon stock should have assurance that they are not exposed to tuberculosis and other forms of contagion; and this can only be done by placing competent veterinarians on the boards of dairy- and food-inspection. The system of State meat-inspection should also receive our attention, and our efforts should not cease until there is a competent veterinarian placed on every board of health in the State.

Briefly let me allude to the work of the Bureau of Animal Industry and the correlative body the State Experiment Station, which have been steadily adding to our wealth of knowledge and advancement, cutting away the brushwood that obscured and covered up the truths hidden deep in nature's methods. They have investigated the perpetuation of species and kinds, and through their researches the ravages and destructive forces of microbic bodies have been checked and our people have in a manner been saved much loss and hardship that brought disappointment and trial as the fruits of their toil. Better still will be the work of the department in the future, for the establishment of the merit-system of appointment has already increased the efficiency of the service, and it will become the aim and ambition of many of our best men who are fitted for this line of work when they see that merit prevails and that tenure of office depends solely on ability to perform well the services required. One now feels like commending this service to the consideration of all, for it affords great opportunity for developing members of our profession, some of whose abilities and powers might otherwise never be fully brought forth. It will enhance this work in every way, and rich fruits yet to be gathered though the services already rendered by this department stand out more prominently as a true wealth-conserver and safeguard to our people than those of any department of the government. When measured by the relative cost to the immense moneyed interest represented by our live-stock industry, it is beyond question a most useful department of our government. Likewise we should as a society put forth every effort to establish the same system of inspection within our State, and ere long the people will hail the day of its introduction. The department of the State work should also be extended along this line in the interest of the people. Tuberculosis, anthrax, glanders, hog-cholera, and all other contagious diseases are increasing every year with the increase of valuable stock, and it is becoming necessary for a more thorough and careful watch to be kept on these diseases in the interest of public health and the live-stock growers. There is manifestly need of a competent assistant in every county, working under the State Veterinarian, to whom the people may look for protection in these cases of need, and they should work under the merit-system, that the people may be insured with a guarantee of competency. The late Dr. V. F. Atkinson, all honor to his name, the promoter of veterinary work among us, had planned and outlined a system similar to this; but sickness and death prevented the execution of his plans. Let us take up the work where he was compelled to lay it down, and give to the people a service creditable to the profession and an honor to our State.

Another thought that I would have you keep in your minds in these days of patent nostrums, specifics, and agents, which are supposed to dispose of every disease by a simple measure, or a fixed plan of treatment, is that the field of scientific medication and the proper use of drugs will for many years be our chief reliance for success and a measure of our worth in every community. Aconite, belladonna, and cinchona bark, aloes, epsom salt, and linseed oil, saltpetre, sweet spirit of nitre and ergot, have not lost their potency for good, and each has its use, more valuable to-day than ever before, for surely he who has lived in the profession the past ten years must have learned how much better to use drugs. How, when and where to employ these efficient agents and the numerous other more potent ones that are crowding upon us every day demand our earnest attention and close study, that we may reap the full harvest of reward by their use and add to the valuable storehouse of veterinary therapeutics that has for so many hundreds of years served well the aims and purposes of our predecessors in the days when microbes, antitoxins, and serums were unknown quantities. I have alluded to these few features of our profession as they present themselves to my mind to-day, simply to remind you of the fact that the scope of our work is enlarging and will continue to enlarge, at the same time demanding from you greater devotion and a deeper attention in the future. These vitascopic views of our work must surely impress upon you one great need which I have briefly alluded to before, and for which I beg your indulgence in returning for a moment, and I would that I might say something to impress upon you its deep and vital importance to our future well-being. Higher veterinary education is the demand of the hour, and I have only words of praise for those schools so true to our interests that have added so much strength to this work during the past year. The opening of the college year of 1896 brought the richest promise on all sides, and it is this great stride, this promised future, and fruition of our hopes and wishes that I desire to refer to for a moment.

It is our duty as members of a chosen profession to commend publicly those who have in these trying financial times courageously taken this step of advancement. They should command from us, one and all, the most earnest support we can give them, and at all times, with voice and pen, we should ever be ready to aid them in this laudable work. In your community you should each take an active interest in any man who may be considering the veterinary profession as his future calling, and see that, so far as you may be able, he shall be so advised and influenced as to select one of the many good schools where he may obtain such an education as will equip him well for the work to be done in the future. In every way you can contribute to the thoroughness of this work, you should do so directly and indirectly, and use every opportunity to enable those who are to be our successors and fill our places when we have passed to the great beyond, to be men of well-filled minds and with a storehouse of knowledge that shall make them better able to fill the *rôle* of a veterinarian than you or I have ever been. For many years we have been accustomed to follow in the line marked out for us by the veterinarians in the older countries of Europe; but to-day we note a change in the aspect of affairs, and observe with pride the veterinarians of the old world following the swath we are cutting through the long-neglected and ignored fields of veterinary sanitary medicine, and I would not be doing a justice to the United States Veterinary Medical Association if I did not call your attention to the fact that all this has come about from their untiring efforts in this line, and we must recognize the fact that all State organizations and their members are only auxiliaries to the national society in all questions of national and to a great extent of local interest. While I am not unmindful of the present financial stringency, it is my duty at this time to urge upon you the necessity of using your earliest opportunity and convenience to secure your membership therein.

I ask you all to give the freest expression to your thoughts and ideas during this meeting of our Society, and would call your attention to the fact that this is the haven toward which our annual pilgrimages are made, where we can place our offerings and tribute on the altar of science as tokens of the appreciation of the responsibilities our profession has laid upon our shoulders. With this hurried review of some of the phases of our work I ask your thorough consideration of the best programme, the able work of your efficient Secretary, ever presented for your consideration, instruction and entertainment, which I am sure will add to the wealth of knowledge of every one present, and when we return to our fields of everyday work it will give us a strength and a confidence that can only be appreciated by those who have regularly attended these gatherings. Finally, in summarizing my work in your midst, I wish to say that there could be no more laudable desire felt by any member of our profession than to be called to the chief place in the organization, and thus to represent his profession in a State like ours. For five years I have been honored by this Society in official place: three years as secretary, one year as censor, and one as president, and I cannot lay down the duties of office without expressing my sincere appreciation of the honors you have conferred upon me. Equally have I felt the great responsibility that rested upon my shoulders, and I am not unmindful of how far short I have fallen in advancing your work through this organization. I shall carry to the end of life pleasant memories of this recognition of your esteem and lasting recollections of the warm support and assistance I have always received from the members of this Society.

